# Experiences and meanings of trans men about breastfeeding in light of the Theory of Social Representations

**DOI:** 10.1590/1980-220X-REEUSP-2024-0006en

**Published:** 2024-10-21

**Authors:** Danilo Martins Roque Pereira, Leonardo Morjan Britto Peçanha, Ednaldo Cavalcante de Araújo, Anderson Reis de Sousa, Danielle Laet Silva Galvão, Monique de Freitas Gonçalves Lima, Maurício Caxias de Souza, Kelly Cordeiro de Carvalho Torres

**Affiliations:** 1 Universidade Federal de Pernambuco, Programa de Pós-Graduação em Enfermagem, Recife, PE, Brazil.; 2 Fundação Oswaldo Cruz, Instituto Nacional de Saúde da Mulher, da Criança e do Adolescente Fernandes Figueira, Flamengo, RJ, Brazil.; 3 Universidade Federal da Bahia, Escola de Enfermagem, Salvador, BA, Brazil.; 4 Universidade Federal de Pernambuco, Programa de Pós-Graduação em Saúde da Criança e do Adolescente, Recife, PE, Brazil.; 5 Lumos Cultural. São Paulo, São Paulo, SP, Brazil.

**Keywords:** Men’s Health, Pregnancy, Social Representation, Sexual and Gender Minorities, Breastfeeding, Salud del Hombre, Embarazo, Representación Social, Minorías Sexuales y de Género, Lactancia Materna

## Abstract

**Objective:**

To analyze the experiences and meanings attributed by trans men to the breastfeeding process in light of the Theory of Social Representations.

**Method:**

A qualitative, descriptive and exploratory study, carried out nationwide, with an intentional sample of five trans men who experienced breastfeeding and aged 18 or over. A semi-structured interview script was applied as a data collection instrument, using the Google Meet^®^ platform, from September to October 2021. The data were described, integrating them with content analysis, resulting in two analytical categories.

**Results:**

Trans men in labor were willing to breastfeed their child, anchoring their representations of breastfeeding in the idea that it is a phenomenon performed exclusively by women and also recognizing this phenomenon as an event that constitutes their masculinity, in addition to describing it as a pedagogical and learning experience with the objective of guaranteeing the healthy development of children.

**Conclusions:**

This study may promote reflections for changing nursing and health practices in different care settings, especially during prenatal care, childbirth and the postpartum period.

## RESUMEN

**Objetivo:**

Analizar las experiencias y significados atribuidos por hombres trans al proceso de lactancia a la luz de la Teoría de las Representaciones Sociales.

**Método:**

Estudio cualitativo, descriptivo y exploratorio, realizado a nivel nacional, con una muestra intencional de cinco hombres trans que han experimentado la lactancia y tienen 18 años o más. Se aplicó como instrumento de recolección de datos la guía de entrevista semiestructurada, utilizando la plataforma Google Meet^®^, de septiembre a octubre de 2021. Se describieron los datos integrándolos con el análisis de contenido, dando como resultado dos categorías analíticas.

**Resultados:**

Los hombres trans en trabajo de parto estaban dispuestos a amamantar a su hijo, anclando sus representaciones sobre la lactancia en la idea de que es un fenómeno realizado exclusivamente por mujeres y también reconocer este fenómeno como un acontecimiento que constituye su masculinidad, además de describirlo como una experiencia pedagógica y de aprendizaje con el objetivo de asegurar el sano desarrollo del niño.

**Conclusiones:**

Este estudio puede promover reflexiones sobre el cambio de las prácticas de enfermería y salud en diferentes entornos de atención, especialmente durante el prenatal, el parto y el posparto.

## INTRODUCTION

Transsexuality refers to an individual and subjective experience in which people do not identify with the gender assigned at birth. “Trans men” can be defined as those individuals who affirm themselves as men socially, regardless of their biological sex, whether or not they require biomedical interventions on their bodies, such as masculinizing mastectomy surgery or total hysterectomy, and who can maintain their reproductive organs viable and have preserved reproductive capacity, and who can express the desire to become pregnant and to conduct breastfeeding processes^(1,2)^.

Some of these individuals who maintain their uterus and breasts may become pregnant, give birth and choose whether or not to breastfeed, requiring specialized support during perinatal and postpartum care. Scientific evidence has already shown that pregnant trans men report feelings of invisibility, isolation and loneliness in prenatal and obstetric care environments that are constituted by the “cisheteronormative” logic, such as “maternity wards”. The lack of inclusive spaces and the presence of professionals who reproduce transphobic discourses constitute barriers to access and violation of rights, going against what is advocated by the Brazilian National Policy for Comprehensive Health Care for Lesbians, Gays, Bisexuals, Transvestites and Transsexuals (LGBT)^(3)^.

Regarding lactation, although trans men and women have breast tissue, the word “breast” is still strongly associated with the female gender. However, for trans women, despite not being able to become pregnant, the development of breasts through breast tissue formation, which is histologically and radiologically indistinguishable from that of cisgender women, allows them to effectively breastfeed their children, which is an important marker of feminization^(4,5)^.

It is important to note that trans men who have undergone masculinizing mammoplasty surgery may have difficulty breastfeeding via the thoracic route, since a large part of the mammary glands is removed, and those who still have breasts may feel uncomfortable during this process, attributed to “intense discomfort” due to being close to the symbol of “femininity”. Therefore, we can state that successful breastfeeding transcends sociocultural, physical and psychological boundaries^(6)^.

The Theory of Social Representations (TSR) of Serge Moscovici and his followers provides an approach to understanding people’s knowledge based on the concrete experience of a specific social group, such as trans men who have had the experience of breastfeeding. This enables healthcare professionals to guide their care, taking into account these individuals’ specific needs, with the aim of transforming their practices towards equitable, comprehensive and effective care^(7)^.

It is worth noting that the TSR is a useful theoretical framework for understanding how people construct and share meanings about certain social phenomena, contributing to the understanding of research data. In this regard, the TSR may enable the understanding of how trans men may have varied perceptions about breastfeeding based on their experiences and cultural contexts and analyze how individuals interpret and attribute meaning to breastfeeding practices or even identify consensuses (shared beliefs) and divergences (divergent beliefs) among research participants on the study topic^(7)^. Thus, discussing breastfeeding by trans men makes it possible to “share new beliefs, knowledge and ways of perceiving the breastfeeding process that are essential in the care relationship”^(8)^.

Although the literature highlights the fact that pregnancy among trans men has had media visibility in recent years and has been included in the agenda of discussions on sexual and reproductive rights, and that healthcare professionals have become more informed about the healthcare required by trans people, there is a scarce number of national and international studies that address the topic or that describe the experiences of breastfeeding by trans men, especially insufficient in the field of nursing^(3)^.

Therefore, it is necessary to conduct this study to address the gaps in the scientific literature and to support advanced nursing practice in order to consider the uniqueness of each person, the recognition of sexual and gender diversity in the context of sexual and reproductive health, and respect for differences in care, especially during the breastfeeding process among trans men. Given the above, this study has as its guiding question: what are the experiences and meanings attributed by trans men to the breastfeeding process? The objective was to analyze the experiences and meanings attributed by trans men to the breastfeeding process in light of the TSR.

## METHOD

### Study Design

This is a qualitative study, structured according to the *Consolidated criteria for Reporting Qualitative research* (COREQ) guidelines.

### Study Location

The current study was conducted with participants associated with prominent organizations dedicated to the LGBT community. The institutions included were the Brazilian National LGBTI+ Alliance, the Patrícia LGBT Outpatient Clinic Gomes, located in Recife, and the LGBT Comprehensive Health Care Coordination of the state of Pernambuco. The choice of these locations took into account the importance of gathering and recognizing this population of interest, meeting the necessary criteria to compose the sample and conduct the research.

### Study Participants

The intentional sample for this study consisted of five trans men, after including trans men who experienced breastfeeding and were 18 years of age or older. Trans men who experienced breastfeeding with cognitive dysfunctions that would hinder participation in the study, who did not have technological resources (computer, cell phone, *tablet, internet*, among others), making it impossible to carry out the research in the context of the pandemic, were excluded. The selection of trans men was based on criteria of convenience and availability. The choice for this approach was motivated by the need to engage the population of interest, since this is a group that is difficult to identify^(9)^. An “invitation letter” was sent to representatives of reference institutions for the LGBT population requesting support and clarifying the research objective.

### Empirical Information Production

Data collection took place from September to October 2021, using the technique of individual interviews with a semi-structured script, virtually, via *Google Meet* ^®(10)^. An objective questionnaire was also applied to characterize the sample. The use of the semi-structured interview technique allowed for a more comprehensive acquisition of information, since it allowed the formulation of new questions based on participants’ responses. This had a positive impact on empirical data collection for this study^(11)^.

A semi-structured interview script was composed of the following guiding questions: tell me about your experience during childbirth and postpartum; tell me about the healthcare provided by the healthcare team during childbirth and postpartum; if you chose to breastfeed postpartum, tell me about this experience. During the interviews, participants were asked to activate the camera on their cell phones or computers, allowing images and recordings of the interviews to be captured using the functionality offered by *Google Meet* ^®^. After this stage was completed, the interviews were transcribed in their entirety and made available to study participants, in order to enable content review and correction, allowing them to make adjustments to the text.

### Data Analysis

The content analysis technique was applied, aiming to analyze the data, which organizes information into pre-analysis, material exploration, treatment of results, inference and interpretation^(12,13)^. Data analysis was anchored by the TSR of Serge Moscovici and followers, as it offers a theoretical basis for understanding the everyday life of specific social groups^(7)^. It gives meaning, direction and guidance to these groups, constituting practical knowledge, both because it is integrated into the experience, which includes a historical, cultural and spatial context, and because it influences people’s communications and behaviors. Thus, it enables the nursing field to better understand social phenomena, through a constructivist approach and direct care and guide healthcare provision based on the recognition of trans men’s particularities and singularities.

### Ethical Aspects

This study was approved by the Federal University of Pernambuco (UFPE) Research Ethics Committee, under Opinion 4.862.503, approved in 2021, in accordance with Resolution 466/12 of the Brazilian National Health Council (BNHC). Participants were informed about the potential risks, benefits and procedures involved in the study, as described in the Informed Consent Form (FICF) and the Image and Testimonial Release and Consent Form, which were made available online through Google Forms^®^. All information that could identify participants was anonymized, using self-declared pseudonyms at the time of data collection, thus ensuring that participation in the study was completely voluntary and spontaneous.

## RESULTS

The central findings of this study are presented based on participant characterization and two analytical categories composed of the narratives of the trans men investigated, anchored in the social representations framework about the phenomenon investigated. It is noteworthy that all participants became pregnant and chose to breastfeed after the birth of their child, during the postpartum period, in a period prior to the completion of this research. Chart 1 shows the study participant characterization.

**Chart 1:** Study participant characterization – Recife, PE, Brazil, 2023.

Identification	Participant characterization
I1 Bernardo	Bisexual transgender man, 26 years old, white, single, father of an only child, unplanned pregnancy through vaginal sex, breastfed immediately after birth, lives in the state of Pernambuco, Brazil.
I2 Iago	Bisexual transgender man, 31 years old, white, in a stable union (transcentered relationship), became pregnant through vaginal sex, father of an only child, used masculinizing hormone during unplanned pregnancy and interrupted it after its confirmation, chose to breastfeed immediately after birth, lives in the state of Pernambuco, Brazil.
I3 Lessa	Heterosexual transgender man, 54 years old, white, in a stable union, father of one biological child and two non-biological children from another relationship of his current wife, unplanned pregnancy through vaginal sex, opted for breastfeeding immediately after birth, lives in the state of Rio de Janeiro, Brazil.
I4 Brandon	Heterosexual transgender man, 25 years old, black, father of an only child, stable union marital relationship (transcentered relationship), planned pregnancy through vaginal sex, chose to breastfeed immediately after birth, lives in the state of Bahia, Brazil.
I5 Azevedo	Pansexual transgender man, 25 years old, black, father of an only child, stable marital relationship, unplanned pregnancy through vaginal sex, chose to breastfeed immediately after birth, lives in the state of Minas Gerais, Brazil.

The findings were structured into thematic categories of analysis, which explain the meanings and experiences of breastfeeding by trans men. In this way, two categories were created, framed within the TSR, based on the elements that compose it:

*Category 1: Breast discomfort and feelings and experiences about breastfeeding*

Category 1 relates to reports of discomfort with the body, especially with the breasts, when engorged with milk during pregnancy and after childbirth. The processes of adaptation to “leaving oneself aside” and “being willing” to provide food for the child, which would be a “greater good”, are also evident.

After the start of breastfeeding, meanings are attributed to this experience as “satisfactory”, “a rewarding moment”, “affective connection” and “learning”. Moreover, difficulties present during the process of human breastfeeding are noted, such as pain, cracked nipples, among others, as well as the importance of the nursing team in care and guidance during this process:

*(...) what screamed the most was the breast... for me it was the worst... having to deal with what I like the least, not to say I hate it, but what I liked the least was seeing it growing and drawing more and more attention to something I hid so much. It was really hard to deal with and then use the breast to breastfeed... it was a lot of work of “putting myself aside for a greater good”, you know? And putting yourself aside, even if it’s for your own child, is something really hard to do. I wouldn’t breastfeed again. But... you know the moment when a child is breastfeeding, and then they look deep into your eyes, you can understand exactly what they’re feeling and you understand that they’re thanking you, you know? They stroke your back, so those little moments were worth it. That moment was gratifying. (I1)*

*(...) breastfeeding was a very complicated thing, because when I had her, I couldn’t breastfeed right away, and I was anxious about it. I tried to learn by myself right from the beginning... the nurse helped me breastfeed my daughter and hold her in my arms. The other father always said, “Are you going to breastfeed?”, and I would answer, “I will, I want to breastfeed”... and he... “Yes, but doesn’t that bother you?”... and I would answer, “It won’t stop me from being more or less of a man... it’s for her own good (the daughter), I have to breastfeed... so how can I refuse?”. Breastfeeding has a learning curve, because for me, it was very difficult in the first few moments alone, right? When I had the support of my father or this nurse, it was super great, but when I was alone, I was scared because you have to hold a breast that size with two little fingers and a child... feeding was very complicated for me... (...). (I2)*

*(...) when he started to stimulate, and then my breasts got huge and I had a lot of milk, I breastfed him, he slept and still had milk to express, to give to other children. I think that the fact of being someone else’s food outweighs everything. The negative thing is the pain when the nipple cracks... what excruciating pain... it seems that the more cracked it is, the hungrier they are. I said, “I can’t take it”. There was a time when it started to bleed, and then I took it off one and put it on the other... I put an ointment that I don’t remember the name of anymore, but then the pain went away when you saw how beautiful and strong he was becoming, how well he was feeding... but it was a pain that made you want to pee... do everything with this pain. (I3)*

*(...) at first, I wasn’t willing to breastfeed. I was willing to go through the pregnancy process, but for personal reasons, because I didn’t have a good relationship with that part of my body, I preferred not to breastfeed... that beforehand, right? It’s an individual process... from that tranquility I had during pregnancy, I was able, for instance, to realize that I was breastfeeding later, and I breastfed for four months. I only stopped breastfeeding because I felt this need to get my hormones back on track, but if it weren’t for that, if we had studies, for instance, that told me that it was safe, that I could get hormonal and breastfeed at the same time, I would most likely have breastfed for a little longer. It was essential for the nursing techniques to teach me how to latch on, right? It’s an extremely painful process, but it’s a very intense emotional connection of feeling that you’re satisfying that baby and that they came from you, that they’re part of you. At three months, I stopped exclusively breastfeeding and started sharing breastfeeding and formula... he only breastfed me twice a day. (I4)*

*Category 2: Home isolation and use of gender-affirming technologies during breastfeeding*

Category 2 refers to how trans men also reproduce a perception that “large” breasts during breastfeeding are still related to an exclusive phenomenon performed by cisgender women. For this reason, feelings of “discomfort” with this part of the body are observed, causing breastfeeding to be restricted to the home environment in the postpartum period, suggesting that it is also a protection strategy against transphobic situations in public places. Gender-affirming technologies, such as bras, become an option for these men when going out to public places, such as work, during the postpartum period, as reported below:

*(...) breastfeeding was really complicated. The only time I left the house and had to breastfeed on the street was in an Uber. I thought it would be the safest place, but the driver’s comments and expression when I was breastfeeding were terrible, and after that, I decided that I would never go out again while breastfeeding. I basically didn’t leave the house for seven months. I had to start wearing a bra again, and seeing myself in that condition, wearing a bra again, felt like my body was slapping me in the face and telling me that I wasn’t who I was showing myself to be. My dysphoria was really active during that period. I worked until about twenty days before my son was born and I would wear a brace that was as tight as possible... it hurt a lot. (I1)*

*(...) breastfeeding was a shock for me. I couldn’t breastfeed in other public places, like on the street, that kind of thing. It was only at home. And if I had to go to other places, I would use the technique of expressing milk and putting it in a bottle, but he stopped doing that pretty quickly. So, that kind of relieved me. It was a lot easier for me. I always deprived myself, I didn’t like to show it off very much, no. (I5)*

Aiming to identify social representations about breastfeeding and the implications for healthcare practice, Chart 2 is presented.

**Chart 2:** Trans men’s social representations about breastfeeding and implications for healthcare practice – Recife, PE, Brazil, 2023.

Social representations	Implications for healthcare practice
Breastfeeding as a guarantee for the healthy development of children	Highlights the benefits of human milk, indicating the importance of promoting health education spaces on breastfeeding during reproductive planning, prenatal and postpartum periods.
Breastfeeding seen as a “satisfying”, “gratifying”, “affective” and “learning” experience	Practice that goes beyond the nutritional value of human milk to meet children’s basic needs, indicating the importance of including pregnant trans men in groups of pregnant individuals during prenatal care, enabling the sharing of experiences and the empowerment of these people.
Breastfeeding seen as something “feminine”	Practice related to the “maternal” social function and performed exclusively by cisgender women; in this case, it may indicate the need to stop milk production in the puerperium among trans men who have breasts. Therefore, if it is necessary to perform a clinical assessment of the breasts, their authorization should be requested during appointments, respecting their autonomy.
Breastfeeding seen as a constituent of masculinity	Practice that breaks with cisheteronormative logic, indicating the need for qualified listening during obstetric care about the possibility of breastfeeding after birth, in promoting spaces that welcome sexual and gender diversity in different care settings and in respecting people’s subjectivity and uniqueness as a condition for well-being.

## DISCUSSION

This study sought to analyze the experiences and meanings attributed by trans men to the breastfeeding process in light of the TSR. It is important to highlight the historical, political and generational context in which the men who participated in the research found themselves, since they make up a population group that has demanded that the healthcare system, whether public or private, fully meet their needs, through recognition of their gender identity, contradicting the cisheteronormative logic in force in the structuring of Brazilian society, as well as its institutions and departments, which include provision of services in the health sector. Therefore, they need to be taken into account in the way the findings are interpreted, as well as in their incorporation into daily professional practices, which is a challenge to be overcome by healthcare professionals^(14)^.

It was observed that trans men were willing to breastfeed their children and represented breastfeeding as a guarantee for the healthy development of their children, even with the subjective and particular difficulties in carrying it out. This representation is anchored in the medical public health discourse that highlights the benefits of human milk, such as its contribution to the person’s immune defense^(15)^.

The ideas about representations of breastfeeding are anchored in a traditional discourse that relates this process to an exclusively “feminine” function, to the point of stating that “oneself is put aside” to exclusively guarantee the feeding of one’s child, which expresses, in a certain way, a “social thought”, as Moscovici points out in the TSR^(7)^. This points to the need for nursing and healthcare professionals to consider and legitimize the experiences of men who wish to breastfeed, strengthening the value of identity of “transpaternity”. However, only one of the study participants breaks with this hegemonic logic and anchors his representation based on the recognition of this phenomenon as an event that constitutes his masculinity.

A clear finding of the delay in meeting trans men’s reproductive health needs in Brazil is the fact that spaces intended for childbirth began a process of qualification, through professional qualification and development of assistance technologies, for caring for pregnant trans people between 2022 and 2023, with the creation of the first “Pregnant Man’s Booklet”, to be published by a Brazilian maternity hospital, located in Salvador, Bahia, being one of these initiatives^(16,17)^. In view of this, it is recommended that the social constructions of transgender masculinities be taken into consideration in the contexts of prenatal care, labor and birth, puerperium and childcare, as well as understanding the biographical ruptures in transmasculine identities resulting from stigmatization^(18,19)^.

The benefits of breastfeeding in strengthening the bond with children are well known, and it even contributes to reducing postpartum depression. However, in a “paternal pregnancy”^(20)^, marked by interruption of continuous testosterone use, return of physical characteristics seen socially as “feminine”, intrinsic to the pregnancy cycle and due to various rights violations, can generate significant impacts on mental health and contribute to the emergence of this condition^(2,21,22,23)^.

It is in this context that situations such as interruption of exclusive breastfeeding, which is recommended until the newborn (NB) is six months old, through the early provision of milk formula as a source of infant nutrition, may become one of the options among this group, since the aforementioned behavior may be related to the need of these men to maintain “masculine” body standards through the use of hormones, as reported in one of the cases in this study, with a view to improving their quality of life^(24,25,26,27)^. Hence, the TSR is useful for understanding that human beings are thinkers and are processing what is around them, whose representation of the collective contributes to individuals’ knowledge^(7)^.

The importance of nurses’ role in the health education process is highlighted with regard to guidance on the risks and benefits of breastfeeding for trans men who have not undergone the surgical procedure of bilateral masculinizing mastectomy, throughout the pregnancy-puerperal cycle so that they can reflect and have the autonomy to decide whether or not they will exclusively breastfeed^(24,25)^.

At the same time, trans men in labor report the awakening of an “affective bond” with their child during breastfeeding, characterizing this practice as a phenomenon that goes beyond the nutritional value of human milk to meet the NB’s basic needs and highlighting the strong relationship established at this unique moment in their lives. This perception is in line with the meanings attributed by cisgender women, who state that the interaction between mother and baby during breastfeeding makes them feel safer and nurtures this dyad of meanings^(28)^. It is worth mentioning, however, reports of families who chose to use a bottle instead of breastfeeding and who, in some way, establish a bond through daily care for children^(6)^.

The fact that pregnancy was not planned, in most cases, may be related, in some way, to greater difficulty in breastfeeding and early weaning, since many individuals report difficulty in “accepting pregnancy” and, consequently, “allowing breastfeeding”, as it may expose their “breasts” and cause discomfort, especially when done in public places. In this regard, it is essential to promote subjects’ autonomy in choosing whether or not to breastfeed, providing support for comprehensive care during this process, as well as enabling discussion about the possibility of pregnancy among trans men and transmasculine people in reproductive planning services^(3,29)^. For these reasons, it is essential that institutions organize their care, management, teaching/training processes for human resources to meet the demands of trans men in the context of breastfeeding^(20)^.

The literature says that some trans men who undergo masculinizing mammoplasty surgery before pregnancy may produce milk, with edema occurring at the site of the surgical scar, with breast growth returning or even reaching the size prior to surgery, and others may not present edema or lactation^(6)^. This scenario may be increasingly present in countries like Brazil, requiring technical/technological development in nursing, in order to satisfactorily respond to the demands related to lactation, as evidenced by research in pharmacy, in terms of human milk’s contribution to trans men’s and children’s mental health^(28)^.

Hence, the “periareolar” surgical approach, in which the nipples remain intact, appears to be one of the best options for those who wish to breastfeed^(6)^. Although milk production may be compromised by surgery, if trans men decide to breastfeed, they should be encouraged to provide their child with as much milk as possible and be informed about the indications, types and methods of supplementation and the need for close monitoring by a healthcare professional^(25,26,27,28,29)^.

It is important to note that, after birth, the return of hormone therapy through the use of testosterone can interfere with the hormones necessary for milk production, such as prolactin, although the use of this substance appears to be safe, because it is not significantly excreted through milk and does not have an immediate effect on the NB. However, it is necessary to observe signs of precocious puberty in children; for this reason, it is recommended to stop using testosterone when pregnancy is detected and during breastfeeding, and this is a recommendation to be carried out during prenatal and postpartum care^(3,30)^.

Discomfort with the appearance of breasts engorged with milk was seen as a “shock” or something “difficult” and “complicated” by three of the five participants in this study. This aspect, seen as negative, is anchored in the socially established idea that larger breasts are a feminine characteristic. In this subjective conflict, these trans men reproduce a discourse permeated by the logic of hegemonic masculinity and use strategies to hide this part of the body, such as wearing coats, in order to avoid having their breasts visible, especially in places with a large circulation of people^(3)^.

The use of a bra, as a “gender affirmation” technology, can be used as a strategy to maintain the chest in a shape closer to that of a “male”, even in the face of possible physical discomfort due to the presence of breasts full of milk. This behavior also represents an individual protection strategy to avoid being socially recognized as a “pregnant or parturient trans man”, given the risk of transphobic violence in public spaces. In this regard, it is important to highlight the need to develop public policies that can raise awareness among the population about the specificities of trans men who become pregnant, covering the areas of health, education, work, transportation and other aspects of daily life^(3)^.

This reflection justifies the need for trans men to increasingly resort to masculinizing mammoplasty after weaning, since there is a greater likelihood of greater discomfort with the body after this moment, and this can generate significant impacts on their mental health^(31)^. Pain and cracked nipples, recurring complications during breastfeeding, have been reported as negative aspects and can also contribute to the introduction of other foods during the recommended period for exclusive breastfeeding or even to the suspension of this process^(14)^. Furthermore, it calls the attention of nursing and healthcare professionals to reflect on how trans men’s trajectory is anchored in reproductive experiences and the exercise of fatherhood, considering the processes of transformation of knowledge towards everyday life, in the face of the strangeness and disturbance that produces influence on people^(7)^.

Considering the issues that emerged from the data on trans men’s social representations of breastfeeding, it is important to highlight the key concepts of “objectification” (creating a scenario, making it familiar) and “anchoring” (attribution of value, interpretation and comparison), present in the TSR, since they are relevant for categorization, labeling, classification, naming or representation of thoughts regarding a trans man’s experiences^(3)^.

Therefore, healthcare for an effective breastfeeding process must begin with prenatal care, in order to discuss the person’s desires, fears and doubts about this phenomenon as well as infant feeding options in case a person wishes not to breastfeed. It is important that the health unit and those who are part of it can offer a welcoming environment, develop a relationship of trust and respect, and provide spaces for continuing education so that professionals recognize the health demands of trans men in the context of breastfeeding.

Primary Health Care (PHC), as a space conducive to prenatal care, should encourage the participation of pregnant trans men in breastfeeding support group meetings, as they may experience feelings of isolation and loneliness during pregnancy^(3)^. Given the bodily changes intrinsic to the pregnancy-puerperal cycle and the possibility of transphobic violence, the presence of a multidisciplinary team in monitoring these people’s mental health is essential^(32,33,34)^. Furthermore, employing professional conduct that recognizes the intersectional sensitivity presented in trans men’s experience is an effective possibility of reducing social segregation and discrimination and strengthening public policies that guarantee social justice^(35)^.

It is important to highlight that the exercise of sexuality is part of sexual and reproductive rights, with the aim of having or not having a pregnancy, with whom and when, as well as its spacing. Most of reports from trans men in this study who became pregnant in an unplanned manner make us reflect on the difficulty in receiving healthcare for these people, especially regarding reproductive planning in PHC, making it impossible for these individuals to access safe contraceptive methods, as needed, and to qualified information, thus exposing them, once again, to greater risk and vulnerability^(3)^.

This study has limitations, since data collection was carried out via Google Meet^®^ as a strategy to prevent COVID-19, which made it impossible to observe the daily lives of participants. However, this study allowed us to elucidate trans men’s social representations about breastfeeding, which may promote reflections for changing nursing and health practices in different care settings, especially during prenatal care, labor and delivery, and the postpartum period.

## CONCLUSION

Trans men’s social representations about breastfeeding refer to it as something “feminine”, to the point of stating that they “set themselves aside” to exclusively ensure the feeding of their child, with movements that break with the hegemonic logic, towards the recognition of breastfeeding as an event that constitutes their construction of masculinity. Thus, it pointed to the awakening of an “affective connection” with their child during breastfeeding, characterizing this practice as a phenomenon that goes beyond the nutritional value of human milk, to meet the NB’s basic needs, but the strengthening of the affective relationship established in the framework of life – breastfeeding -, attributing values to an experience seen as “satisfactory”, “gratifying”, “affective” and “learning”. Given the nature and specificity of the findings found in this study, a discussion is suggested in light of the gender and sexuality study theoretical framework, which is a limitation of this research.
