# Supplementary material for: Experiences and meanings of trans men about breastfeeding in light of the Theory of Social Representations
Source: Rev Esc Enferm USP. 2024 Oct 21;58:e20240006. doi: 10.1590/1980-220X-REEUSP-2024-0006en (PMC11499245; doi:10.1590/1980-220X-REEUSP-2024-0006en)
Supplement: Translation

# Translation: Experiências e significados de homens trans sobre amamentação à luz da Teoria das Representações Sociais

**DOI:** 10.1590/1980-220X-REEUSP-2024-0006pt

**Authors:** Danilo Martins Roque Pereira, Leonardo Morjan Britto Peçanha, Ednaldo Cavalcante de Araújo, Anderson Reis de Sousa, Danielle Laet Silva Galvão, Monique de Freitas Gonçalves Lima, Maurício Caxias de Souza, Kelly Cordeiro de Carvalho Torres

**Affiliations:** 1 Universidade Federal de Pernambuco, Programa de Pós-Graduação em Enfermagem, Recife, PE, Brasil.; 2 Fundação Oswaldo Cruz, Instituto Nacional de Saúde da Mulher, da Criança e do Adolescente Fernandes Figueira, Flamengo, RJ, Brasil.; 3 Universidade Federal da Bahia, Escola de Enfermagem, Salvador, BA, Brasil.; 4 Universidade Federal de Pernambuco, Programa de Pós-Graduação em Saúde da Criança e do Adolescente, Recife, PE, Brasil.; 5 Lumos Cultural. São Paulo, São Paulo, SP, Brasil.

**Keywords:** Saúde do Homem, Gravidez, Representação Social, Minorias Sexuais e de Gênero, Aleitamento Materno

## Abstract

**Objetivo:**

Analisar as experiências e significados atribuídos por homens trans ao processo de amamentação à luz da Teoria das Representações Sociais.

**Método:**

Estudo qualitativo, descritivo e exploratório, realizado em âmbito nacional, com uma amostra intencional de cinco homens trans que experienciaram a amamentação e com idade igual ou superior aos 18 anos. Aplicou-se o roteiro de entrevista semiestruturado como instrumento de coleta de dados, utilizando a plataforma *Google Meet* ^®^, de setembro a outubro de 2021. Os dados foram descritos, integrando-os à análise de conteúdo, resultando em duas categorias analíticas.

**Resultados:**

Os homens trans parturientes se dispuseram a amamentar sua criança, ancorando suas representações sobre a amamentação na ideia de que se trata de um fenômeno desempenhado, exclusivamente, por mulheres e reconhecendo, também, esse fenômeno como um acontecimento que constitui sua masculinidade, além de descrevê-la como uma experiência pedagógica e de aprendizado com o objetivo de garantir o desenvolvimento saudável da criança.

**Conclusões:**

Este estudo poderá promover reflexões para a mudança da prática em enfermagem e saúde nos diversos cenários do cuidado, especialmente durante a assistência pré-natal, ao parto e ao puerpério.

## INTRODUÇÃO

A transexualidade diz respeito a uma experiência individual e subjetiva em que pessoas não se reconhecem com o gênero atribuído no momento do nascimento. Pode-se definir enquanto “homens trans” aqueles indivíduos que se afirmam como homens socialmente, independentemente de sexo biológico, necessitando ou não de intervenções biomédicas corpóreas, a exemplo da cirurgia de mastectomia masculinizadora ou histerectomia total, podendo manter seus órgãos reprodutivos viáveis e com capacidade reprodutiva preservada, e podem manifestar o desejo pela gestação e a condução dos processos de amamentação^(1,2)^.

Alguns desses sujeitos que mantêm o útero e mamas podem gestar, parir e optar ou não pela amamentação, necessitando de um apoio especializado durante os cuidados perinatais e puerperais. Evidências científicas já demonstraram que homens trans grávidos relatam sentimentos de invisibilidade, isolamento e solidão em ambientes de cuidados pré-natal e obstétrico constituídos pela lógica “cisheteronormativa”, a exemplo das “maternidades”. A falta de espaços inclusivos e a presença de profissionais que reproduzem discursos transfóbicos se constituem barreiras de acesso e de violação de direitos, indo na contramão do que preconiza a Política Nacional de Atenção Integral à Saúde de Lésbicas, Gays, Bissexuais, Travestis e Transexuais (LGBT)^(3)^.

No que diz respeito à lactação, apesar de os homens e as mulheres trans possuírem tecido mamário, a palavra “mama” ainda se associa fortemente ao gênero feminino. No entanto, para mulheres trans, apesar de não poder engravidar, o desenvolvimento das mamas por meio da constituição de um tecido mamário, que é histológica e radiologicamente indistinguível das mulheres cisgênero, oportuniza estas a amamentar de forma eficaz suas crianças, sendo esse um importante marcador de feminização^(4,5)^.

Ressalta-se que homens trans que realizaram o procedimento cirúrgico de mastectomia masculinizadora podem ter dificuldades para amamentar por via torácica, uma vez que ocorre a retirada de grande parte das glândulas mamárias, e os que ainda possuem mamas poderão se sentir desconfortáveis durante esse processo, atribuído a um “desconforto intenso”, por estar próximo do símbolo da “feminilidade”. Com isso, podemos afirmar que o sucesso da amamentação perpassa fronteiras socioculturais, físicas e psicológicas^(6)^.

A Teoria das Representações Sociais (TRS) de Serge Moscovici e seus seguidores proporciona uma abordagem para compreender o conhecimento das pessoas com base na experiência concreta de um grupo social específico, como é o caso dos homens trans que passaram pela experiência da amamentação. Isso possibilita que os profissionais de saúde possam orientar seu cuidado, levando em consideração as necessidades específicas desses indivíduos, com o objetivo de transformar suas práticas em direção a uma assistência equitativa, abrangente e eficaz^(7)^.

Destaca-se que a TRS é um quadro teórico útil para compreender como as pessoas constroem e compartilham significados sobre determinados fenômenos sociais, contribuindo para o entendimento dos dados de uma pesquisa. Nesse sentido, a TRS poderá possibilitar a compreensão de como homens trans podem ter percepções variadas sobre a amamentação baseadas em suas experiências e contextos culturais e analisar como os indivíduos interpretam e atribuem significado às práticas de amamentação ou até mesmo identificar consensos (crenças compartilhadas) e divergências (crenças divergentes) entre os participantes da pesquisa sobre a temática do estudo^(7)^. Assim, discutir sobre a amamentação por homens trans possibilita “compartilhar novas crenças, saberes e formas de perceber o processo de amamentar que são imprescindíveis na relação de cuidado”^(8)^.

Embora a literatura destaque o fato de que a gravidez entre homens trans tem tido uma visibilidade midiática nos últimos anos e ter sido inserida na pauta das discussões sobre os direitos sexuais e reprodutivos, bem como profissionais de saúde tenham se tornado mais informados sobre os cuidados à saúde demandados por pessoas trans, há um número escasso de estudos nacionais e internacionais que abordem a temática ou que descrevam as experiências de amamentação por homens trans, especialmente insuficientes no campo da enfermagem^(3)^.

Assim, faz-se necessário realizar este estudo para responder às lacunas existentes na literatura científica e subsidiar uma prática avançada de enfermagem de forma a considerar a singularidade de cada pessoa, o reconhecimento da diversidade sexual e de gênero no âmbito da saúde sexual e reprodutiva e o respeito às diferenças no cuidado, especialmente durante o processo de amamentação entre homens trans. Diante do exposto, este estudo tem como pergunta condutora: quais as experiências e significados atribuídos por homens trans ao processo de amamentação? Objetivou-se analisar as experiências e significados atribuídos por homens trans ao processo de amamentação à luz da TRS.

## MÉTODO

### Tipo de Estudo

Trata-se de estudo qualitativo, estruturado nas diretrizes do *Consolidated criteria for Reporting Qualitative research* (COREQ).

### Local de Estudo

O estudo atual foi conduzido com participantes associados a organizações de destaque dedicadas à comunidade LGBT. As instituições incluídas foram a Aliança Nacional LGBTI+, o Ambulatório LGBT Patrícia Gomes do Recife e a Coordenação de Atenção Integral à Saúde LGBT de Pernambuco. A escolha desses locais levou em consideração a importância de reunir e reconhecer essa população de interesse, atendendo aos critérios necessários para compor a amostra e conduzir a pesquisa.

### Participantes do Estudo

A amostragem intencional deste estudo se constitui por cinco homens trans, após estabelecidos os seguintes critérios de inclusão: homens trans que experienciaram a amamentação e com idade igual ou superior a 18 anos; e critérios de exclusão: homens trans que experienciaram a amamentação com disfunções cognitivas que prejudicassem a participação no estudo; que não possuíam recursos tecnológicos (computador, celular, *tablet*, *Internet*, entre outros), impossibilitando a realização da pesquisa diante do contexto de pandemia. A seleção dos homens trans se deu por critérios de conveniência e disponibilidade. A opção por essa abordagem foi motivada pela necessidade de engajar a população de interesse, uma vez que trata-se de um grupo de difícil identificação^(9)^. Foi enviada uma “carta convite” aos representantes das instituições de referência para a população LGBT solicitando apoio e esclarecendo o objetivo da pesquisa.

### Produções de Informações Empíricas

A coleta de dados aconteceu de setembro a outubro de 2021, utilizando a técnica de entrevistas individuais com roteiro semiestruturado, de forma virtual, pela plataforma *Google Meet* ^®(10)^. Realizou-se, também, a aplicação de um questionário objetivo para caracterização da amostra. A utilização da técnica de entrevista semiestruturada permitiu uma aquisição mais abrangente de informações, uma vez que possibilitou a formulação de novas perguntas com base nas respostas dos participantes. Isso teve um impacto positivo na coleta de dados empíricos para este estudo^(11)^.

Um roteiro de entrevista semiestruturado foi composto pelas seguintes questões norteadoras: fale-me sua experiência durante o momento do parto e pós-parto; fale-me como foi sua assistência à saúde prestada pela equipe de saúde durante o parto e pós-parto; caso tenha optado por amamentar no pós-parto, fale-me como se deu esta experiência. No decorrer das entrevistas, os participantes foram solicitados a ativar a câmera de seus celulares ou computadores, permitindo a captura de imagens e gravações das entrevistas por meio da funcionalidade oferecida pela plataforma *Google Meet* ^®^. Após a conclusão dessa etapa, as entrevistas foram transcritas na sua totalidade e disponibilizadas aos participantes do estudo, a fim de possibilitar a revisão e correção do conteúdo, permitindo que fizessem ajustes no texto.

### Análise de Dados

Aplicou-se a técnica de análise de conteúdo, objetivando analisar os dados, que organiza as informações em pré-análise, exploração do material, tratamento dos resultados, inferência e interpretação^(12,13)^. A análise dos dados foi ancorada pela TRS de Serge Moscovici e seguidores, uma vez que oferece uma base teórica para a compreensão da vida cotidiana de grupos sociais específicos^(7)^. Ela confere significado, direção e orientação a esses grupos, constituindo um conhecimento prático, tanto por estar integrada na experiência, que inclui um contexto histórico, cultural e espacial, quanto por influenciar as comunicações e comportamentos das pessoas. Assim, possibilita que a área da enfermagem melhor compreenda os fenômenos sociais, por meio de uma abordagem construtivista e direcione o cuidado e oriente a prestação da assistência em saúde com base no reconhecimento das particularidades e singularidades dos homens trans.

### Aspectos Éticos

Este estudo foi aprovado pelo Comitê de Ética em Pesquisa da Universidade Federal de Pernambuco (UFPE), sob Parecer n^o^. 4.862.503, aprovado no ano de 2021, em conformidade com a Resolução n^o^. 466/12 do Conselho Nacional de Saúde (CNS). Os participantes foram informados sobre os potenciais riscos, benefícios e procedimentos envolvidos no estudo, conforme descritos no Termo de Consentimento Livre e Esclarecido (TCLE) e no Termo de Autorização para Uso de Imagem e Depoimento, que foram disponibilizados *online* por meio da plataforma *Google Forms* ^®^. Todas as informações que pudessem identificar os participantes foram anonimizadas, utilizando pseudônimos autodeclarados no momento da coleta de dados, assegurando, assim, que a participação no estudo fosse totalmente voluntária e espontânea.

## RESULTADOS

Os achados centrais deste estudo estão apresentados a partir da caracterização dos participantes e de duas categorias analíticas compostas pelas narrativas dos homens trans investigados, ancorados no quadro de representações sociais acerca do fenômeno investigado. Destaca-se que todos os participantes engravidaram e optaram pela amamentação após o nascimento da criança, durante o pós-parto, em período anterior à realização desta pesquisa. Dispõe-se no Quadro 1 a caracterização dos participantes do estudo.

**Quadro 1:** Caracterização dos participantes do estudo – Recife, PE, Brasil, 2023.

Identificação	Caracterização dos participantes
E1 Bernardo	Homem transexual bissexual, 26 anos, branco, solteiro, pai de filho único, gravidez não planejada por meio de sexo vaginal, realizou a amamentação no pós-parto imediato, reside no estado de Pernambuco, Brasil.
E2 Iago	Homem transexual bissexual, 31 anos de idade, branco, relação conjugal de união estável (relação transcentrada), engravidou por meio de sexo vaginal, pai de filho único, fez uso de hormônio masculinizante durante a gravidez não planejada e o interrompeu após sua confirmação, optou pela amamentação no pós-parto imediato, reside no estado de Pernambuco, Brasil.
E3 Lessa	Homem transexual heterossexual, 54 anos, branco, relação conjugal de união estável, pai de um filho biológico e dois não biológicos de outra relação da atual esposa, gravidez não planejada por meio de sexo vaginal, optou pela amamentação no pós-parto imediato, reside no estado do Rio de Janeiro, Brasil.
E4 Brandon	Homem transexual heterossexual, 25 anos de idade, preto, pai de filho único, relação conjugal de união estável (relação transcentrada), gravidez planejada por meio de sexo vaginal, optou pela amamentação no pós-parto imediato, reside no estado da Bahia, Brasil.
E5 Azevedo	Homem transexual pansexual, 25 anos, preto, pai de filho único, relação conjugal de união estável, gravidez não planejada por meio de sexo vaginal, optou pela amamentação no pós-parto imediato, reside no estado de Minas Gerais, Brasil.

Os achados encontrados foram estruturados em categorias temáticas de análise, as quais explicitam os significados e experiências da amamentação por homens trans. Desse modo, originaram-se duas categorias, enquadradas na TRS, a partir dos elementos que a compõem:

*Categoria 1: Desconforto com as mamas e os sentidos e experiências sobre a amamentação*

A categoria 1 relaciona-se aos relatos de desconforto com o corpo, especialmente com as mamas, quando ingurgitadas de leite durante a gestação e após o parto. Evidenciam-se, também, os processos de adaptação ao “deixar-se de lado” e “se dispor” para prover a alimentação da criança, que seria um “bem maior”.

Após o início da amamentação, são atribuídos sentidos a essa experiência como “satisfatória”, “momento gratificante”, “ligação afetiva” e de “aprendizado”. Além disso, constatam-se as dificuldades presentes durante o processo de aleitamento humano, a exemplo das dores, rachaduras nos mamilos, entre outros, bem como a importância da equipe de enfermagem no cuidado e orientação durante esse processo:

*(...) o que mais gritou foi a mama… foi para mim o pior... ter que lidar com o que menos gosto, para não dizer que ode, mas o que eu menos gostei foi ver aquilo crescendo e cada vez chamando mais atenção para algo que eu escondia tanto. Foi bem complicado lidar e usar a mama depois para amamentar… foi muito um trabalho de “estou me deixando de lado para um bem maior”, sabe? E se deixar de lado, mesmo que seja para um filho seu, é algo bem complicado de se fazer. Eu não amamentaria de novo. Mas... sabe o momento que a criança está mamando, e aí ele olha no fundo do seu olho, você consegue entender exatamente o que ele está sentindo e você entende que ele está agradecendo, sabe? Ele faz um carinho nas suas costas, então esses pequenos momentos valiam à pena. Esse momento foi gratificante. (E1)*

*(...) foi uma coisa bem complicada essa questão da amamentação, porque, quando eu a tive, não pude amamentar logo, e eu estava ansioso por isso. Eu tentei aprender sozinho logo de início... a enfermeira que me ajudou a amamentar minha filha e a colocar ela no colo. O outro pai sempre dizia: “Você vai amamentar?”, e eu respondia: “Vou, quero amamentar”... e ele... “Sim, mas isso não te incomoda?”... e eu respondia: “Não vai me deixar de ser mais ou menos homem... é para o bem dela (a filha), eu tenho que amamentar... então, como é que eu vou me negar?”. A amamentação tem significado de aprendizado, porque, para mim, foi muito difícil nos primeiros momentos sozinho, né? Quando eu tinha o apoio do pai ou dessa enfermeira, era super ótimo, mas quando eu estava sozinho, eu ficava com medo porque você segurar uma mama desse tamanho com dois dedinhos e uma criança ... foi muito complicado a alimentação para mim… (...). (E2)*

*(...) quando começou a estimular, e aí a mama ficou imensa e eu tinha muito leite, eu o amamentava, ele dormia e ainda tinha leite para tirar, para dar a outras crianças. Eu acho que o fato de ser alimento de outra pessoa supera qualquer coisa. O negativo é a dor que dá quando o bico da mama racha... que dor desesperadora... parece que, quanto mais rachado, mais fome eles têm. Eu falei: “Eu não vou aguentar”. Teve uma época que chegou a sangrar, e aí tira de um e bota no outro... passei uma pomada que eu não me lembro mais o nome, mas aí a dor passava quando você vê como ele estava ficando bonito e forte, como ele estava se alimentando bem..., mas era uma dor que dá vontade de mijar... fazer tudo com essa dor. (E3)*

*(...) no começo, eu não estava disposto a amamentar, eu estava disposto a passar pelo processo de gestação, mas, por questões pessoais minhas, por não ter uma boa relação com essa parte do meu corpo, eu preferi não passar pela amamentação... isso previamente, né? É um processo individual… a partir dessa tranquilidade que eu tive durante a gestação, eu consegui, por exemplo, me perceber depois amamentando, e amamentei durante quatro meses. Só não amamentei mais sendo por conta que eu sentia essa necessidade de voltar com meus hormônios, mas, se não fosse isso, se nós tivéssemos estudos, por exemplo, que me dissessem que é seguro, que eu me hormonizaria e amamentaria ao mesmo tempo, muito provavelmente eu teria amamentado por um pouco mais de tempo. Foi fundamental as técnicas de enfermagem ensinarem a pega, né? É um processo extremamente doloroso, mas é uma ligação afetiva muito intensa de você sentir que está satisfazendo aquele bebê e que ele saiu de você, que é parte sua. Com três meses, eu parei de fazer só a amamentação exclusiva e comecei a dividir a amamentação e fórmula... ele só mamava umas duas vezes por dia em mim. (E4)*

*Categoria 2: Isolamento domiciliar e uso de tecnologias de afirmação de gênero durante a amamentação*

A categoria 2 se refere a como os homens trans, também, reproduzem uma percepção de que as mamas “grandes” durante o aleitamento humano ainda se relacionam enquanto um fenômeno exclusivo e desempenhado por mulheres cisgênero. Por este motivo, verificam-se os sentimentos de “desconforto” com essa parte do corpo, fazendo com que a amamentação seja restrita ao meio domiciliar no pós-parto, sugerindo, inclusive, tratar-se, também, de uma estratégia de proteção diante de situações de transfobia em locais públicos. As tecnologias de afirmação de gênero, a exemplo do sutiã, tornam-se uma opção na saída desses homens para locais públicos, a exemplo do trabalho, durante o puerpério, conforme relatos abaixo:

*(...) amamentar foi bem complicado, assim. A única vez que eu saí de casa que eu precisei amamentar na rua foi dentro do Uber. Eu achei que seria o local mais seguro, sendo que aí o comentário e a cara do motorista, quando eu estava amamentando, foram péssimos, e, depois disso, eu decidi que não ia mais sair enquanto eu estivesse amamentando. Foram sete meses basicamente sem sair de casa. Eu tive que voltar a usar sutiã, e me ver naquela condição, usando sutiã de novo, parecia que era o meu corpo dando tapa na minha cara e dizendo que eu não era aquilo que eu estava me mostrando ser. As disforias ficaram bem ativadas nesse período. Eu trabalhei até uns vinte dias antes de meu filho nascer e eu ia com colete apertado no máximo... doía muito. (E1)*

*(...) a amamentação foi um baque para mim. Amamentar em outros lugares públicos, como na rua, essas coisas, eu não conseguia. Era só dentro de casa. E se eu tivesse que ir para outros lugares, usava a técnica de tirar o leite e colocar na mamadeira, mas até que ele largou bem rápido. Então, isso meio que já me aliviou. Foi bem mais fácil para mim. Eu sempre me privei, não gostava muito de mostrar, não. (E5)*

Objetivando a identificação das representações sociais sobre a amamentação e as implicações para a prática do cuidado em saúde, segue o Quadro 2.

**Quadro 2:** Representações sociais de homens trans sobre a amamentação e as implicações para a prática do cuidado em saúde – Recife, PE, Brasil, 2023.

Representações sociais	Implicações para a prática do cuidado em saúde
Amamentação como garantia para o desenvolvimento saudável da criança	Ressalta os benefícios do leite humano, indicando a importância da promoção de espaços de educação em saúde sobre a amamentação durante o planejamento reprodutivo, pré-natal e puerpério.
Amamentação vista como experiência “satisfatória”, “gratificante”, “afetiva” e de “aprendizado”	Prática que ultrapassa o valor nutricional do leite humano para suprir as necessidades básicas da criança, indicando a importância da inclusão de homens trans grávidos em grupos de gestantes durante o pré-natal, possibilitando o compartilhamento de experiências e o empoderamento dessas pessoas.
Amamentação vista como algo “feminino”	Prática relacionada à função social “materna” e desempenhada, exclusivamente, por mulheres cisgênero; nesse caso, pode indicar a necessidade da interrupção da produção de leite no puerpério entre os homens trans que possuem mamas. Assim, caso seja necessário realizar a avaliação clínica das mamas, deve-se solicitar a autorização destes durante os atendimentos, respeitando sua autonomia.
Amamentação vista como constituinte de sua masculinidade	Prática que rompe com a lógica cisheteronormativa, indicando a necessidade da escuta qualificada durante a assistência obstétrica sobre a possibilidade de amamentar após o nascimento, na promoção de espaços de acolhimento da diversidade sexual e de gênero nos diversos cenários de cuidado e no respeito à subjetividade e singularidade das pessoas como condição para o bem-estar.

## DISCUSSÃO

Este estudo buscou analisar as experiências e significados atribuídos por homens trans ao processo de amamentação à luz da TRS. Importa destacar o contexto histórico, político e geracional em que os homens que participaram da pesquisa se encontravam, uma vez que compõem um grupo populacional que tem demandado do sistema de saúde, quer seja público ou privado, o atendimento integral das suas necessidades, mediante o reconhecimento de sua identidade de gênero, contrariando a lógica cisheteronormativa vigente na estruturação da sociedade brasileira, bem como de suas instituições e departamentos, que incluem a prestação de serviços no setor da saúde. Portanto, necessitam ser levados em consideração na maneira como interpretar os achados, bem como incorporá-los ao cotidiano das práticas profissionais, sendo esse um desafio a ser superado pelos profissionais de saúde^(14)^.

Observou-se que os homens trans se dispuseram a amamentar sua criança e representaram a amamentação como uma garantia para o desenvolvimento saudável de seus filhos, mesmo com as dificuldades subjetivas e particulares em ealiza-la. Essa representação ancora-se no discurso médico sanitarista que ressalta os benefícios do leite humano, a exemplo de sua contribuição na defesa imunológica da pessoa^(15)^.

As ideias sobre as representações acerca da amamentação se ancoram em um discurso tradicional que relaciona esse processo a uma função, exclusivamente, “feminina”, a ponto de afirmar “deixar-se de lado” para garantir, exclusivamente, a alimentação de sua criança, o que expressa, de certa forma, um “pensamento social”, conforme aponta Moscovici na TRS^(7)^. Isto posto, aponta para a necessidade de que profissionais de enfermagem e saúde considerem e legitimem as experiências de homens que desejam desempenhar a amamentação, fortalecendo a valorização da identidade da “transpaternidade”. Contudo, apenas um dos participantes do estudo rompe com essa lógica hegemônica e ancora sua representação a partir do reconhecimento desse fenômeno como um acontecimento que constitui sua masculinidade.

Uma constatação evidente do retardo do atendimento às necessidades de saúde reprodutiva dos homens trans no Brasil é o fato de que espaços destinados à realização de partos iniciaram um processo de habilitação, por meio da qualificação profissional e elaboração de tecnologias assistenciais, para o atendimento de pessoas trans gestantes entre os anos de 2022 e 2023, sendo a criação da primeira “Caderneta do Gestante”, a ser publicada por um hospital maternidade brasileiro, localizado em Salvador, Bahia, uma dessas iniciativas^(16,17)^. Diante disso, recomenda-se que as construções sociais das masculinidades transgêneras sejam levadas em consideração nos contextos de assistência ao pré-natal, parto e nascimento, puerpério e puericultura, bem como entendendo as rupturas biográficas nas identidades transmasculinas decorrentes da estigmatização^(18,19)^.

Sabem-se os benefícios da amamentação no fortalecimento do vínculo com a criança, contribuindo, inclusive, para a diminuição da depressão pós-parto. No entanto, em uma “gestação paterna”^(20)^, marcada pela interrupção do uso contínuo da testosterona, o retorno de características físicas vistas socialmente como “femininas”, intrínsecas ao ciclo-gravídico e por diversas violações de direitos, pode gerar impactos significativos na saúde mental e contribuir para o surgimento desse agravo^(2,21–23)^.

É nesse contexto que situações como a interrupção da amamentação exclusiva, que é preconizada até os seis meses de vida do recém-nascido (RN), por meio da oferta precoce de fórmula láctea como fonte de alimentação infantil, podem passar a ser uma das opções entre esse grupo, uma vez que o comportamento supracitado pode relacionar-se com a necessidade desses homens em manter os padrões corpóreos “masculinos” por meio do uso de hormônios, como relatado em um dos casos deste estudo, com vistas à melhoria de sua qualidade de vida^(24–27)^. Nessa direção, a TRS é útil para entender que o ser humano é pensante e encontra-se em processamento do que está à sua volta, cuja representação do coletivo contribui para o conhecimento do indivíduo^(7)^.

Ressalta-se a importância do papel do enfermeiro no processo de educação em saúde no que diz respeito às orientações dos riscos e benefícios da amamentação para os homens trans que não realizaram o procedimento cirúrgico da mastectomia bilateral masculinizadora, durante toda a assistência ao ciclo gravídico-puerperal, para que possam refletir e tenham autonomia de decidir se irão ou não realizar a amamentação exclusiva^(24,25)^.

Ao mesmo tempo, homens trans parturientes relatam o despertar de uma “ligação afetiva” junto à sua criança durante a amamentação, caracterizando essa prática como um fenômeno que ultrapassa o valor nutricional do leite humano para suprir as necessidades básicas do RN e dando destaque à forte relação estabelecida nesse momento único de sua vida. Essa percepção vai de encontro com os significados atribuídos por mulheres cisgênero, afirmando que a interação genitora-bebê durante a amamentação faz com que se sintam mais seguras e nutre esse binômio de significados^(28)^. Cabe citar, porém, relatos de famílias que optaram pelo uso de mamadeira à amamentação na mama e que, de alguma maneira, estabelecem a vinculação por meio do cuidado com a criança no dia a dia^(6)^.

O fato de a gravidez não ter sido planejada, na maioria dos casos, pode relacionar-se, de alguma maneira, a uma maior dificuldade em amamentar e no desmame precoce, uma vez que muitos indivíduos relatam a dificuldade em “aceitar a gestação” e, consequentemente, “permitir a amamentação”, pois poderão expor as “mamas” e ocasionar um desconforto, especialmente quando for realizada em locais públicos. Nesse sentido, faz-se imprescindível a promoção da autonomia do sujeito na escolha ou não em amamentar, fornecendo subsídios para a integralidade do cuidado durante esse processo, bem como possibilitar a discussão sobre a possibilidade de gestação entre homens trans e pessoas transmasculinas nos serviços de planejamento reprodutivo^(3,29)^. Por essas razões, é imprescindível que as instituições organizem seus processos assistenciais, gerenciais, de ensino/formação de recursos humanos para o atendimento das demandas dos homens trans no contexto da amamentação^(20)^.

A literatura diz que alguns homens trans que realizam a cirurgia de mastectomia masculinizadora antes da gestação podem produzir leite, ocorrendo um edema em local da cicatriz cirúrgica, observando-se a volta do crescimento da mama ou até mesmo atingindo o tamanho anterior à cirurgia, e outros podem não apresentar edema ou lactação^(6)^. Esse cenário poderá ser cada vez mais presente em países como o Brasil, sendo necessário o desenvolvimento técnico/tecnológico no âmbito da atuação em enfermagem, a fim de responder satisfatoriamente às demandas voltadas à lactação, como evidenciou investigação da área da farmácia, em termos da contribuição do leite humano na saúde mental de homens trans e de crianças^(28)^.

Nesse sentido, a abordagem cirúrgica “periareolar”, na qual os mamilos permanecem íntegros, parece ser uma das melhores opções para aqueles que desejam amamentar^(6)^. Ainda que a produção de leite possa ser comprometida pela cirurgia, se os homens trans decidirem amamentar, esses devem ser encorajados a fornecer à criança a maior quantidade possível de leite produzido e ser informados sobre as indicações, os tipos e métodos de suplementação e a necessidade de um acompanhamento rigoroso junto a um profissional de saúde^(25–29)^.

Destaca-se que, após o parto, o retorno da hormonização por meio do uso da testosterona pode interferir nos hormônios necessários para a produção de leite, a exemplo da prolactina, embora o uso dessa substância parece ser seguro, porque não é significativamente excretado por meio do leite e não tem efeito imediato no RN. No entanto, é preciso observar sinais de puberdade precoce na criança; por esse motivo, recomenda-se a interrupção do uso da testosterona quando detectada a gestação e durante a amamentação, sendo essa uma recomendação a ser realizada durante os atendimentos pré-natais e puerperais^(3,30)^.

O desconforto com a aparência da mama ingurgitada de leite foi visto como um “baque” ou algo “difícil” e “complicado” entre três dos cinco participantes deste estudo. Esse aspecto visto como negativo ancora-se na ideia socialmente estabelecida de que as mamas em maior tamanho se configuram enquanto uma característica feminina. Neste conflito subjetivo, esses homens trans reproduzem um discurso permeado pela lógica da masculinidade hegemônica e se utilizam das estratégias para a ocultação dessa parte do corpo, a exemplo da utilização de casacos, objetivando não ter suas mamas evidenciadas, principalmente, em locais de grande circulação de pessoas^(3)^.

O uso do sutiã, enquanto uma tecnologia de “afirmação de gênero”, pode ser utilizado como estratégia para manter o tórax no formato mais próximo do “masculino”, mesmo diante de um possível desconforto físico com a presença de mamas cheias de leite. Esse comportamento traduz, também, uma estratégia de proteção individual para não ser reconhecido socialmente enquanto um “homem trans grávido ou parturiente”, diante dos riscos de ocorrência de violência transfóbica em espaços públicos. Nesse aspecto, destaca-se a necessidade da construção de políticas públicas que possam sensibilizar a população sobre as especificidades de homens trans que engravidam, perpassando o campo da saúde, educação, trabalho, transporte e outros aspectos da vida cotidiana^(3)^.

Essa reflexão justifica a necessidade de homens trans recorrerem, cada vez mais, à mastectomia masculinizadora após o desmame, uma vez que existe uma maior probabilidade de um maior desconforto com o corpo após esse momento e que pode gerar impactos significativos em sua saúde mental^(31)^. As dores e rachaduras nos mamilos, complicações recorrentes durante a amamentação, foram relatadas como aspectos negativos e podem contribuir, também, para a introdução de outros alimentos durante o período recomendado para a amamentação exclusiva ou mesmo à suspensão deste processo^(14)^. Além disso, chama a atenção dos profissionais de enfermagem e saúde a refletirem sobre como encontra-se ancorada a trajetória de homens trans em experiências reprodutivas e do exercício da paternidade, considerando os processos de transformação do conhecimento em direção ao cotidiano, diante do estranhamento e da perturbação que produz influência sobre as pessoas^(7)^.

Diante das questões emergidas dos dados das representações sociais dos homens trans sobre a amamentação, importa destacar os conceitos-chave de “objetivação” (confecção de um cenário, tornando-o familiar) e “ancoragem” (atribuição de valor, interpretação e comparação), presentes na TRS, uma vez que são relevantes para a categorização, rotulação, classificação, nomeação ou representação de pensamentos a respeito das experiências de um homem trans^(3)^.

Portanto, a assistência em saúde para um efetivo processo de amamentação deve iniciar-se desde o cuidado pré-natal, de forma a discutir quais os desejos, medos e dúvidas dessa pessoa sobre esse fenômeno, bem como as opções de alimentação infantil em caso do desejo de não amamentar. É importante que a unidade de saúde e aqueles que a integram possam ofertar um ambiente acolhedor, desenvolver uma relação de confiança e respeito, proporcionando espaços de educação permanente, de forma que os profissionais reconheçam as demandas de saúde de homens trans no contexto da amamentação.

A Atenção Primária à Saúde (APS), enquanto espaço propício para o cuidado pré-natal, deve encorajar a participação dos homens trans grávidos em reuniões de grupos de apoio à amamentação, uma vez que esses podem experimentar sentimentos de isolamento e solidão durante a gestação^(3)^. Diante das mudanças corporais intrínsecas ao ciclo gravídico-puerperal e da possibilidade de ocorrência de violências transfóbicas, é fundamental a presença da equipe multidisciplinar no acompanhamento da saúde mental dessas pessoas^(32–34)^. Além disso, empregar uma conduta profissional que reconheça a sensibilidade interseccional apresentada na experiência dos homens trans é uma possibilidade eficaz de diminuição da segregação e discriminação social e o fortalecimento de políticas públicas que garantam a justiça social^(35)^.

É importante destacar que é parte dos direitos sexuais e reprodutivos o exercício da sexualidade, objetivando ter ou não uma gestação, com quem e quando, bem como seu espaçamento. A maioria dos relatos dos homens trans deste estudo e que engravidaram de modo não planejado faz com que possamos refletir sobre a dificuldade no atendimento à saúde por essas pessoas, em especial sobre o planejamento reprodutivo na APS, impossibilitando o acesso desses indivíduos a métodos contraceptivos seguros, conforme sua necessidade, e a informações qualificadas, dessa forma, expondo-os, mais uma vez, a um maior risco e vulnerabilidade^(3)^.

Este estudo apresenta limitações, uma vez que a coleta foi realizada via plataforma *Google Meet* ^®^ como estratégia à prevenção da COVID-19, o que impossibilitou a observação do cotidiano dos participantes. No entanto, este estudo permitiu elucidar as representações sociais de homens trans sobre a amamentação, que poderão promover reflexões para a mudança da prática em enfermagem e saúde nos diversos cenários do cuidado, especialmente durante a assistência pré-natal, ao parto e nascimento e puerpério.

## CONCLUSÃO

As representações sociais dos homens trans acerca da amamentação referem-se de que se trata de algo “feminino”, a ponto de afirmar “deixar-se de lado” para garantir, exclusivamente, a alimentação de sua criança, com movimentos de rompimento da lógica hegemônica, em direção ao reconhecimento da amamentação enquanto um acontecimento que constitui a sua construção de masculinidade. Destarte, apontou para o despertar de uma “ligação afetiva” junto à sua criança durante a amamentação, caracterizando essa prática como um fenômeno que ultrapassa o valor nutricional do leite humano, para suprir as necessidades básicas do RN, mas o fortalecimento da relação afetiva estabelecida no marco da vida – amamentação -, atribuindo valores a uma experiência vista como “satisfatória”, “gratificante”, “afetiva” e de “aprendizado”. Diante da natureza e especificidade dos achados encontrados neste estudo, sugere-se uma discussão à luz do referencial teórico dos estudos de gênero e sexualidades, sendo essa uma limitação da pesquisa.
